# Supplementary material for: Rapid turnover of life-cycle-related genes in the brown algae
Source: Genome Biol. 2019 Feb 14;20:35. doi: 10.1186/s13059-019-1630-6 (PMC6374913; doi:10.1186/s13059-019-1630-6)
Supplement: Supplementary file 3 — Protein alignments, corresponding Gblocks html file and CDS sequences for the lineage conserved gametophyte-biased genes. (ZIP 2109 kb) [file 13059_2019_1630_MOESM3_ESM.zip › Con_GA_alignments/Ec-20_002100.aa_ali.fasta-gb.htm]

Ec-20\_002100.aa.fasta.trans.aa\_ali.fasta


## Gblocks 0.91b Results

Processed file: **Ec-20\_002100.aa.fasta.trans.aa\_ali.fasta**  
Number of sequences: **2**  
Alignment assumed to be: **Protein**  
New number of positions: **871** (selected positions are underlined in blue)

```
                         10        20        30        40        50        60
                 =========+=========+=========+=========+=========+=========+
Ec-20_002100     MGKTTAEGGDGGNSS-HIRTFLRIRPSKRPSGYIKVDEFDKTRLGFHVPVETKDGEYVNN
MSTRG.23465.1    MGKSAAEGRDGGNSSSHIRTFLRVRPSKRPSGFINVDEYDKTKLGFHVPVETKDGEYVNN
                 ############### ############################################


                         70        80        90       100       110       120
                 =========+=========+=========+=========+=========+=========+
Ec-20_002100     TRTSHKFKFDGVLGEKATQEDVFAKIGKGAVSNVLNGYNSTVFAYGQTGSGKTFTVTGGA
MSTRG.23465.1    TRTSHNFKFDGVLGEKATQEDVFAKIGKGAVYNVLDGYNSTVFAYGQTGSGKTFTVTGGA
                 ############################################################


                        130       140       150       160       170       180
                 =========+=========+=========+=========+=========+=========+
Ec-20_002100     ERYVDRGIIPRALSLIFAEFKERSDQQFTCHISYLEIYNEQGFDLLDPSHDSKKLEDLAK
MSTRG.23465.1    ERYVDRGLIPRALSLIFAEFKERSDQQFTCHVSYLEIYNEQGFDLLDPSHDSKKLEELPK
                 ############################################################


                        190       200       210       220       230       240
                 =========+=========+=========+=========+=========+=========+
Ec-20_002100     VALMEDEDGNIHLRNLSVYLAANEEEALNYLFLGDTNRAISETAMNKASSRSHCIFTISV
MSTRG.23465.1    VALMEDEDGNIHLRNLSVYLAANEEEALNYLFLGDTNRAISETAMNKASSRSHCIFTISV
                 ############################################################


                        250       260       270       280       290       300
                 =========+=========+=========+=========+=========+=========+
Ec-20_002100     EGRKHGSDKVMRSKLHLVDLAGSERVHKTQTGGQTLVEAKHINVSLFFLEMVIVALRERG
MSTRG.23465.1    EGRKHGSEKVMRSKLHLVDLAGSERVHKTQTGGQTLVEAKHINVSLFFLEMVIVALRERG
                 ############################################################


                        310       320       330       340       350       360
                 =========+=========+=========+=========+=========+=========+
Ec-20_002100     AKGRQHIPYRNSMMTSVLRDSLGGNCKTVMIATVNAEAAQTEESISTCRFAQRVSTIKND
MSTRG.23465.1    TKGRQHIPYRNSMMTSVLRDSLGGNCKTVMIATVNAEMAQTDESISTCRFAQRVSTIKNE
                 ############################################################


                        370       380       390       400       410       420
                 =========+=========+=========+=========+=========+=========+
Ec-20_002100     ARVNEDVDPGVLIRRLRSDNATLSDEVAFLKGETGDGEELTSEEAEDLKDRCRAFVEDPA
MSTRG.23465.1    ARVNEDVDPGVLIRRLRSECATLNDEVAFLKGESGDGEELTTEEAEDLKNRCRIFVEDPA
                 ############################################################


                        430       440       450       460       470       480
                 =========+=========+=========+=========+=========+=========+
Ec-20_002100     PSAQLSIGAYTLTKLKRCFSVFKAMVLDGGRALSTGGAHSQGHDMVGEGGPAGAMTKLGE
MSTRG.23465.1    PGAQLSIGAYTLTKLKRCFSVFKAMVVDGRQALMSSNGRSG-----VEGTPHSHGQVLAG
                 ########################################       ###########  


                        490       500       510       520       530       540
                 =========+=========+=========+=========+=========+=========+
Ec-20_002100     GSTGELGGQLLSLRDALRQRDNEIAILVNMLKQGKCHACAAAPSSATPGATT---GGGLP
MSTRG.23465.1    --AGGHAVKLLSLQDALRQRDNEIAILVNMLKQGRCHVCAAANPSAPPATRNRPGKGGIA
                    #############################################        ####


                        550       560       570       580       590       600
                 =========+=========+=========+=========+=========+=========+
Ec-20_002100     PNRQHDPGGENASPQHHNRRSPLPGAAAVGAATGASGGKLLANGEGAGRVVVAPKGTVGG
MSTRG.23465.1    PDRQHDPGGKTATAQSQKTLVPLP-ASAAAAHSLAREASVPVSYMGAGSGGVAPKGTVGG
                 ######################## ##########          ###############


                        610       620       630       640       650       660
                 =========+=========+=========+=========+=========+=========+
Ec-20_002100     RDAGGRHRGGSVSGVERPSDASVLGDPQRAFGYFRERHSGRVALEENKKLLGEKYARAKA
MSTRG.23465.1    RDTGVRFNG-NVGGVAAPSDSKVLGDPQRAFGYFRERHSGKEALEENKKLLGEKYARAKA
                 #########  #################################################


                        670       680       690       700       710       720
                 =========+=========+=========+=========+=========+=========+
Ec-20_002100     MGERVNQARSTIQYLKNTIEQVRRERALDGLMEEGSVGGSKGDDEDRNSRPQVGLGEEED
MSTRG.23465.1    MGERVNQARNTIQYLKNTIEQVRRERALDGLVGDGGRRGD-GEDDDDEER-QMDLDEKED
                 #######################################            #########


                        730       740       750       760       770       780
                 =========+=========+=========+=========+=========+=========+
Ec-20_002100     EEETKCKAAIEVEKRVYKGSFQELRELKSEIEHIQRLLEKGRSKMQADFQAWYEPLACSV
MSTRG.23465.1    EEETKCKAAIEVEKRVYKGSFQELRELKSEIEHIQRLLEKGRSKMQADFQSWYEPLLCSA
                 ############################################################


                        790       800       810       820       830       840
                 =========+=========+=========+=========+=========+=========+
Ec-20_002100     EEEDGAVAS--EGNCALGEGG-TKPKAQS--FG-GGDDKPRGAHHHAPQGHRQAVVGKTT
MSTRG.23465.1    KEEKGVVAQSLARPVHVREAGPDRPQGVSSRHGNGGGGASRAVCDNPSHGNEQVDVLESR
                 ########                          ##########################


                        850       860       870       880       890       900
                 =========+=========+=========+=========+=========+=========+
Ec-20_002100     RERQS--EEPAAFDGS-GKDGANTPTRRPRPPVDAWGTPPDSTSRRRGP--LSSVATGSV
MSTRG.23465.1    LETRSGTGEMPIFGGDKGGGGANTPT-RPRPRLNAWGTPPTSVTHRRGPAALSSAVGHVA
                 #####                      ######################  #########


                        910       920       930       940       950       960
                 =========+=========+=========+=========+=========+=========+
Ec-20_002100     SGGDG----GGSKTESAPTSGGGRGAWHGGAGGGGLLPPLTGNKEADDDIAAFYRAKEEL
MSTRG.23465.1    SDGDDKGSDGGGVSNSASTAGGGAGVLLGAAGGEGVFPPLTGNKEADDDIAAFYRAKKEL
                 ####     ###################################################


                        970
                 =========+=====
Ec-20_002100     ERRKSAAGRNSGGR-
MSTRG.23465.1    ERRKSVAGGGNGVKN
                 ############
```

```
Parameters used
Minimum Number Of Sequences For A Conserved Position: 2
Minimum Number Of Sequences For A Flanking Position: 2
Maximum Number Of Contiguous Nonconserved Positions: 8
Minimum Length Of A Block: 10
Allowed Gap Positions: None
Use Similarity Matrices: Yes
```

```
Flank positions of the 13 selected block(s)
Flanks: [1  15]  [17  460]  [468  478]  [484  528]  [537  564]  [566  575]  [586  609]  [612  699]  [712  788]  [815  845]  [868  889]  [892  904]  [910  972]  

New number of positions in Ec-20_002100.aa.fasta.trans.aa_ali.fasta-gb:  871  (89% of the original 975 positions)
```
